# Familial primary calcific band-shaped keratopathy with late onset systemic disease: a case series and review of the literature

**DOI:** 10.1186/s13256-024-04429-y

**Published:** 2024-03-10

**Authors:** Parisa Abdi, Hassan Asadigandomani, Arman Amirkhani, Sara Taghizadeh, Zohreh Nozarian

**Affiliations:** Translational Ophthalmology Research Center (TORC), Farabi Eye Hospital, Tehran University of Medical Sciences, Qazvin Square, Qazvin Street, Box 14176-13151, Tehran, Iran

**Keywords:** Band shaped keratopathy, DALK, Keratoplasty, Case report

## Abstract

**Background:**

Familial calcific band-shaped keratopathy (BSK) is a very rare disease, with no underlying cause. There is no underlying disease in this form of the disease. This article introduces a family with seven children, three of whom were diagnosed with familial primary calcific BSK. One of them developed a systemic disease 38 years after ocular manifestation.

**Case presentation:**

In this case report, three Iranian siblings from a family with familial calcific band-shaped keratopathy (BSK) are introduced. Systemic and ocular examinations performed on these patients indicated the occurrence of chronic kidney disease in the older child, a 41-year-old woman, 38 years after ocular manifestation. The examinations conducted on the other two siblings revealed no pathological findings. The 41-year-old sister and 37-year-old brother underwent unilateral deep anterior lamellar keratoplasty (DALK), while the 33-year-old sister underwent bilateral superficial keratectomy (SK).

**Conclusion:**

Considering the late onset of systemic disease in one of the siblings diagnosed with familial calcific band-shaped keratopathy (BSK), it is crucial to emphasize the necessity of long-term follow-up for these patients and their families.

## Background

Calcific band-shaped keratopathy (BSK) is a white/gray colored deposit that occurs in the superficial layer of the cornea, which generally manifests in the interpalpebral fissure [1]. These corneal deposits resembling “Swiss cheese” usually consist of calcium hydroxyapatite, non-crystalline phosphate forms, and carbonate salts of calcium [2]. This clinical manifestation is often associated with ocular inflammatory conditions such as chronic inflammation, chronic uveitis, and also systemic diseases, particularly hypercalcemia [1, 3]. In the early stages of the disease, there are generally no specific symptoms. However, as the disease progresses, symptoms such as foreign body sensation, photophobia, epiphora, and decreased visual acuity will occur. In the absence of a known underlying disease, a primary form of the disease exists, which is so rare and generally manifests during the first decade of life, with a progressive course [4]. In this case report, a family with seven children is introduced, three of whom were diagnosed with familial primary calcific BSK and were undergoing follow-up. One of them received a diagnosis of a systemic disease after 38 years of ocular manifestation. Therefore, the importance of continuous systemic surveillance in patients and also their families with familial primary calcific BSK should be considered.

## Case presentation

Two Iranian siblings from a family of nine (which includes seven children) were referred to the Corneal Clinic at Farabi Eye Hospital with complaints of blurred vision in both eyes since childhood. The parents of these children were first cousins. The best corrected visual acuity (BCVA) of these siblings was 1–2/10 according to the Snellen chart. These two siblings, a 41-year-old woman and a 37-year-old man, also mentioned blurred vision and deafness in one of their sisters in the family, while other family members did not complain of any problems.

In the slit lamp examination, evidence of calcific BSK was present in both eyes of the patients. During the next visit, when another sister of the patients (33 years old) also attended, the evidence of bilateral calcific BSK was clear, but the BCVA was much better (4/10 in the right eye and 6/10 in the left eye). As visible, the rest of the ocular examination was unremarkable.

In the review of the documents for the 41-year-old woman, there was evidence of kidney disease, including elevated creatinine levels and proteinuria (indicative of nephrotic syndrome), based on a workup conducted last year. She was under the care of both a rheumatology and nephrology subspecialist for treatment.

Until the patients were referred to our clinic, they had been under regular follow-up since childhood in another center. In each annual follow-up visit, routine ophthalmologic examinations along with laboratory information, including calcium, phosphorus, kidney function tests, electrolytes, and the level of parathyroid hormone (PTH) in the blood, have been examined, and the results have not had any pathological points.

Detailed systemic examinations in other family members were conducted, encompassing assessments of calcium, phosphorus, urea, creatinine, electrolytes, alkaline phosphatase and parathyroid hormone (PTH) levels in the blood. Additionally, urine tests for calcium, phosphorus and creatinine yielded normal results. Musculoskeletal evaluation, chest X-ray, and kidney ultrasound also showed no abnormalities.

**Fig. 1:**
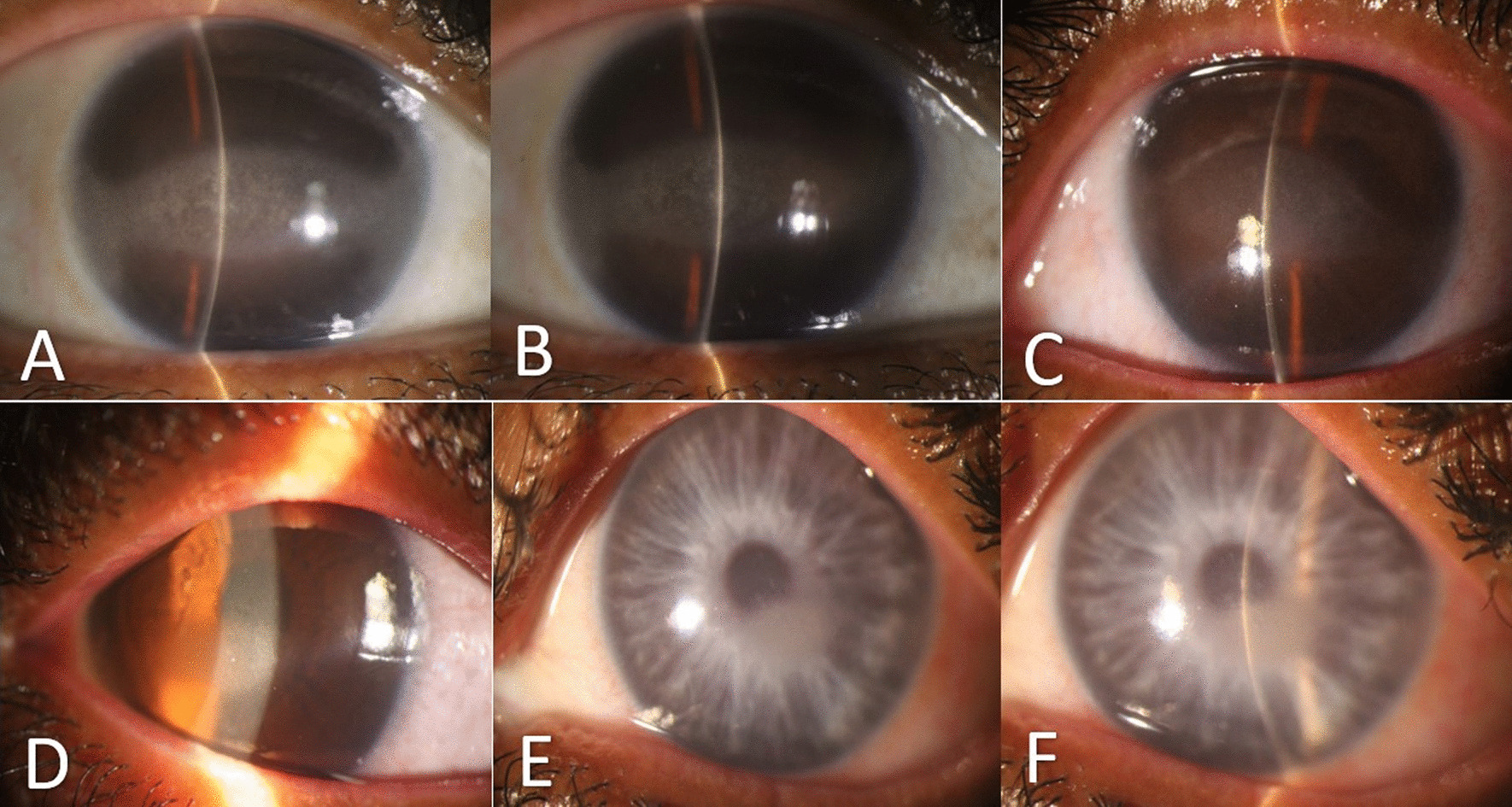
Preoperative slit photographs of three Iranian siblings (**A**, **B** 41 years old sister; OD, OS), (**C**, **D** 37 years old brother; OD, OS), (**E**, **F** 33 years old sister; OD, OS)

**Fig. 2:**
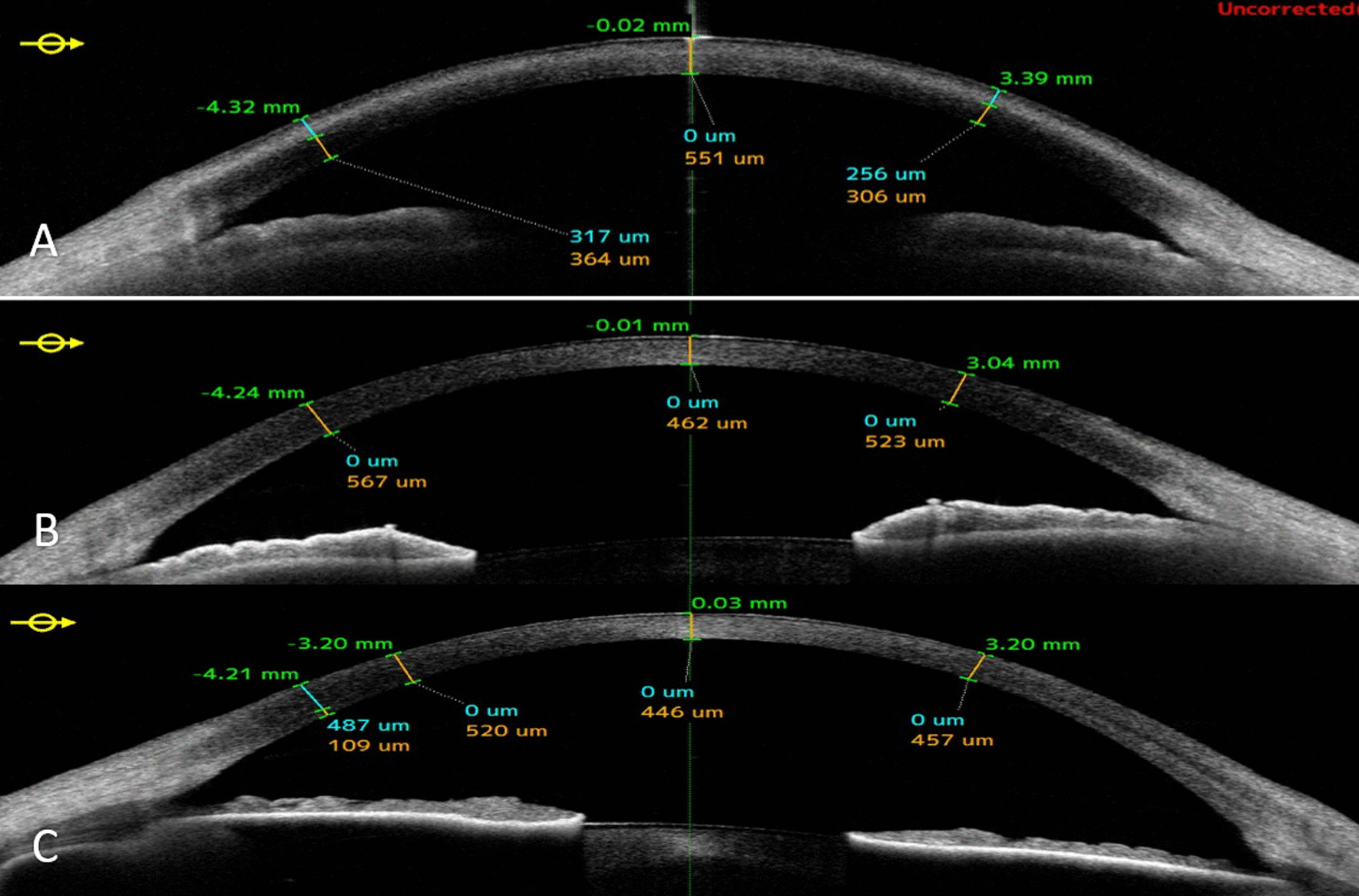
Anterior Segment OCT (AS-OCT) of three Iranian siblings (right eyes); superficial deposition of dens materials (**A** 41 years old sister; OD), (**B** 37 years old brother; OD), (**C** 33 years old sister; OD)

The probable diagnosis for the patients was primary calcific BSK (Fig. 1). An AS OCT was performed, which revealed superficial and dense deposits in line with the areas observed during the examination, confirming the diagnosis (Fig. 2).

**Fig. 3:**
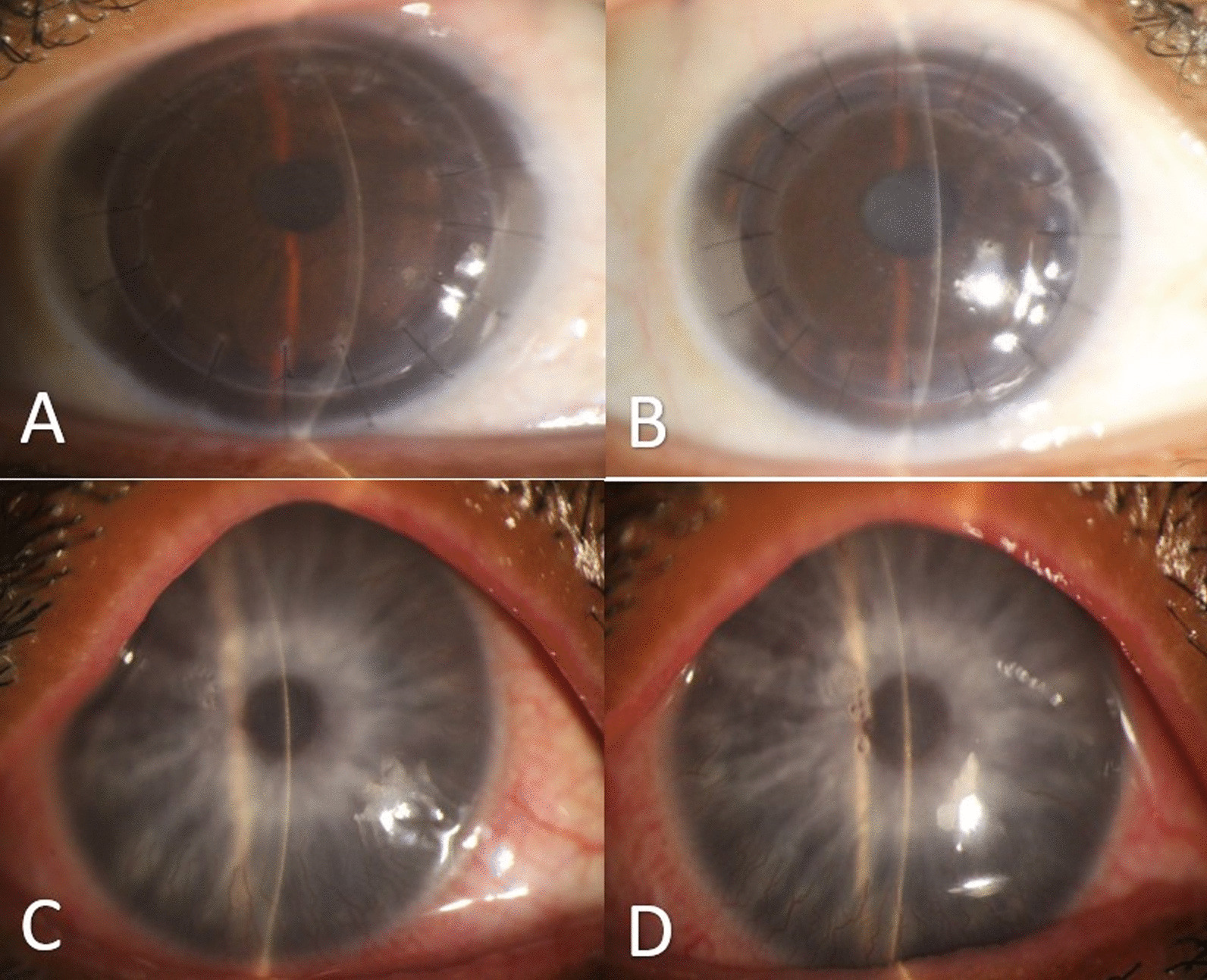
Postoperative slit photos (**A** 41 years old sister; OD), (**B** 37 years old brother; OD), (**C**, **D** 33 years old sister; OD, OS)

Due to reduced vision in the older siblings, it was decided to perform unilateral DALK and they underwent surgery on their right eyes. The younger sister was scheduled for bilateral SK due to the absence of significant blurriness in her vision, with complaints limited to photophobia and foreign body sensation. In the post-operative examination, the older sibling's vision improved to 4/10, and the younger sister's complaints were alleviated (Fig. 3). Six months after the surgeries, the BCVA of the two older siblings reached 4/10 in both eyes and no signs of rejection were observed in the grafts. The BCVA in the younger sister was 6/10 in the right eye and 7/10 in the left eye, and she did not complain of epiphora, photophobia, or foreign body sensation.

**Fig. 4:**
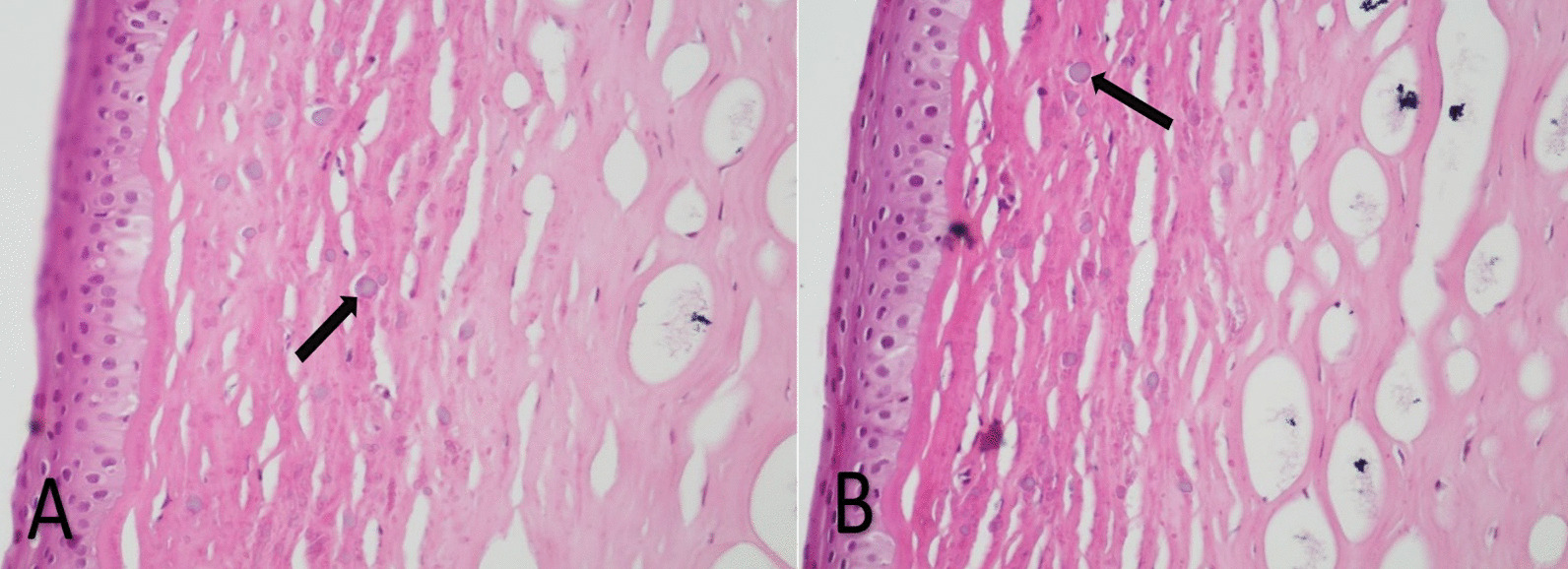
Corneal histopathological slides of patients (magnification × **100**, H/E staining), (**A** 41 years old sister), (**B** 37 years old brother). Arrows show the location of calcium deposits (superficial stroma and extracellularly)

After the surgery, the patient's cornea samples were sent to the pathology department for histopathological examination. As you can see in Fig. 4, the histopathological slides of the patients, calcium deposition is observed in the superficial area of the stroma and also cystic degeneration is observed in the entire thickness of the stroma.

## Discussion

Three siblings with familial calcific BSK have been briefly mentioned in this case report and literature review. Systemic and ocular examinations indicated the occurrence of kidney disease in the older sister (41 years old), 38 years after the appearance of ocular manifestations. The occurrence of systemic disease in the elderly sister several decades after ocular symptoms highlights the importance of regular systemic monitoring in these patients compared to other similar studies that did not associate familial calcific BSK with any underlying disease.

Familial calcific BSK is a very rare disease with very few reports in the literature [4, 5]. This disease occurs in otherwise normal eyes without concurrent pathologies and typically, there are no other systemic or ocular disorders observed in affected patients [6]. Calcific BSK is generally secondary to chronic and inflammatory eye disorders or disruptions in calcium and phosphorus metabolism. The pattern of calcium deposition in these cases can elucidate the underlying cause of the disease. In chronic eye diseases, calcium deposition is extracellular and in the basement membrane of epithelial cells, Bowman's layer, and superficial stroma. Conversely, in general metabolic disorders, calcium deposits occur intracellularly [7]. The pattern of calcium deposition in our patients was in the superficial stroma and extracellularly, which was consistent with the presence of chronic diseases and contrary to the pattern of metabolic disorders.

The most uncommon variant of calcific BSK is the familial form, and the reported cases of this form in the literature are summarized in Table 1.

**Table 1:** Previous case reports of familial calcific BSK

Reported by	Year	Description
Fuchs *et al*. [8]	1939	Two siblings aged 11 (brother) and 13 (sister)
Streiff *et al*. [9]	1946	Three children in an eleven-numbered family (9 children)
Glees *et al*. [10]	1950	A father with his child
Ticho *et al*. [4]	1979	Twelve-year-old sister with a 5-year-old brother
Arora *et al*. [5]	2007	6-year-old sister with 4-year-old brother

If calcific band-shaped keratopathy occurs in first-degree family members without any underlying ocular or systemic factors identified in the investigations, the familial form of the disease may be implicated.

Several genetic disorders, such as NBCe1, have been linked to various forms of familial disease, but this disorder is also associated with proximal renal tubular acidosis [11], which differs from the renal condition observed in the first case.

**Fig. 5:**
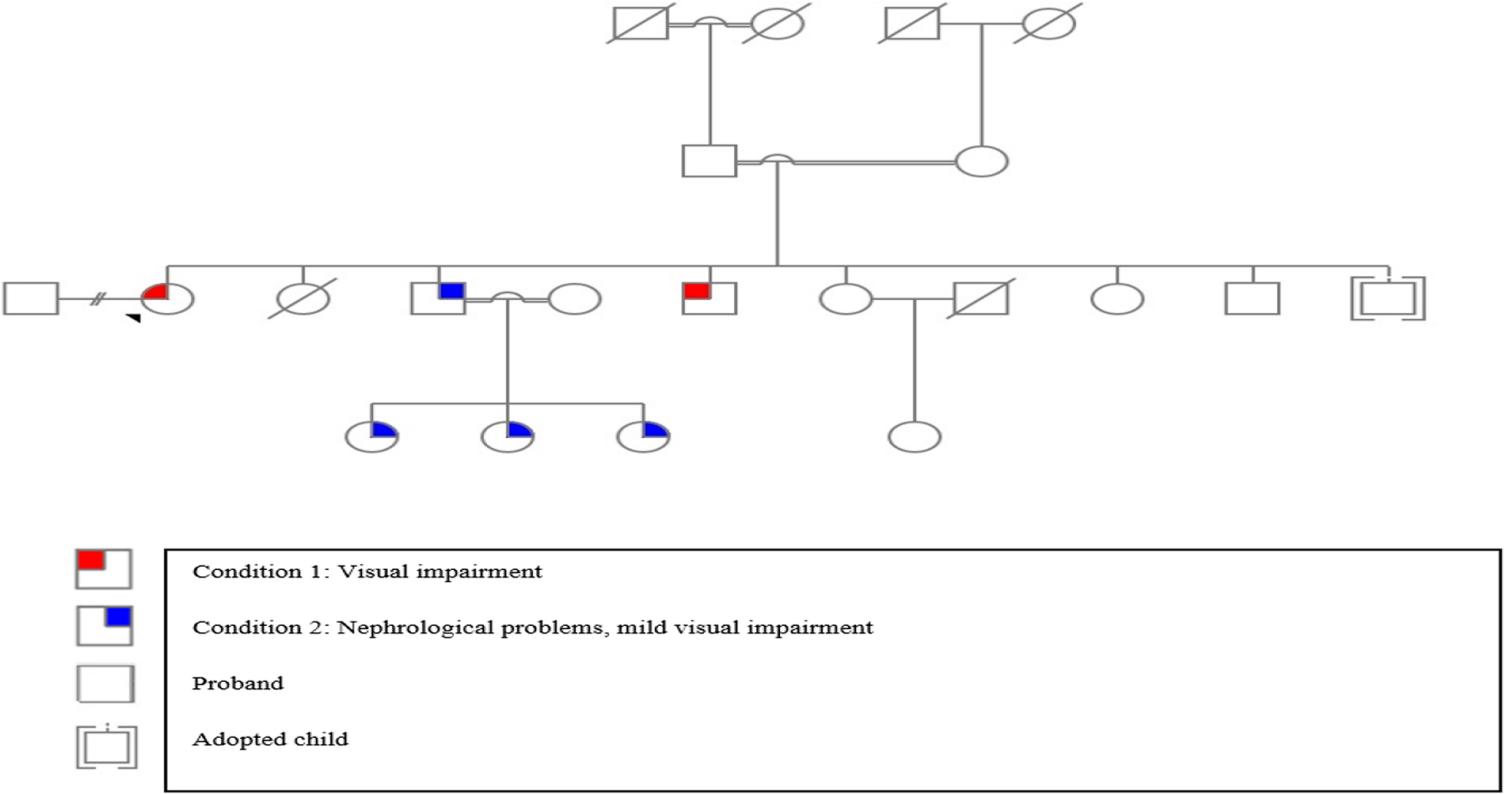
Pedigree of family

According to the pedigree information (Fig. 5), consanguinity, and the recurrence of the disease manifestations in multiple offspring, the likelihood of a hereditary genetic condition is quite high. The most presumptive inheritance pattern is autosomal recessive. Furthermore, the presence of consanguineous marriages and the vertical transmission of the disease within this individual's family may also suggest autosomal dominant inheritance patterns.

After reviewing the patient's medical records related to nephrological assessments and consultations, we found that multiple rheumatological laboratory tests, including ANA, complement level, anti-Ro and anti-La, and anti-ds DNA were conducted repeatedly. Additionally, a kidney biopsy has been performed. However, no definite diagnosis for the patient's nephrological issue has been established. At one point, due to the presence of a malar rash and proteinuria, treatment in the form of prednisolone and hydroxychloroquine was initiated with the provisional diagnosis of systemic lupus erythematosus (SLE). Nevertheless, the laboratory findings and kidney biopsy results (which showed an absence of specific immunofluorescence deposition and only the presence of chronic glomerulonephritis, fibrosis and tubular damage) do not strongly support the diagnosis of SLE.

The significant aspect in the case of the older sister in our report is the onset of nephrological problems in the last three years. Considering that the patient's eye disease began in childhood and the nephrological problems started at the age of 38, it is likely that the patient's eye disease is not caused by the systemic condition. Instead, genetic factors may contribute to the development of both conditions.

While it is highly probable that the familial form of the disease is responsible for this condition within this family, the most crucial point to emphasize is the necessity for continuous and lifelong follow-up of these patients and their families. Over time, another disease, whether related to calcific BSK or not, may appear.

Among the limitations of our study, we must highlight the absence of genetic tests to determine the nature of the disease. According to the family pedigree, the possibility of hereditary conditions, whether autosomal recessive or autosomal dominant, was considered. The only way to definitively diagnose these conditions would have been through a comprehensive genetic sequencing. Unfortunately, due to the financial constraints and the family’s inability to cover the cost, this diagnostic avenue was not pursued.

## Conclusion

Despite familial calcific BSK being a primary disease with no identified underlying ocular or systemic factors, lifelong follow-up is very important due to the potential for systemic diseases related to ocular manifestations.

## Data Availability

The data that support the findings of this study are available on request from the corresponding author.

## Abbreviations

BSK: Band shaped keratopathy
DALK: Deep anterior lamellar keratoplasty
SK: Superficial keratectomy
BCVA: Best corrected visual acuity
PTH: Parathyroid hormone
AS-OCT: Anterior segment optical coherence tomography
OD: Right eye
OS: Left eye
ANA: Anti-nuclear antibody
ds-DNA: Double stranded deoxyribonucleic acid
SLE: Systemic lupus erythematous
